# Intracellular Calcium Spikes in Rat Suprachiasmatic Nucleus Neurons Induced by BAPTA-Based Calcium Dyes

**DOI:** 10.1371/journal.pone.0009634

**Published:** 2010-03-10

**Authors:** Jin Hee Hong, Cheol Hong Min, Byeongha Jeong, Tomoyoshi Kojiya, Eri Morioka, Takeharu Nagai, Masayuki Ikeda, Kyoung J. Lee

**Affiliations:** 1 Center for Cell-dynamics and Department of Physics, Korea University, Seoul, Republic of Korea; 2 Graduate School of Innovative Life Science, Toyama University, Toyama, Japan; 3 Laboratory for Nanosystems Physiology, Research Institute for Electronic Science, Hokkaido University, Sapporo, Japan; Vanderbilt University, United States of America

## Abstract

**Background:**

Circadian rhythms in spontaneous action potential (AP) firing frequencies and in cytosolic free calcium concentrations have been reported for mammalian circadian pacemaker neurons located within the hypothalamic suprachiasmatic nucleus (SCN). Also reported is the existence of “Ca^2+^ spikes” (i.e., [Ca^2+^]_c_ transients having a bandwidth of 10∼100 seconds) in SCN neurons, but it is unclear if these SCN Ca^2+^ spikes are related to the slow circadian rhythms.

**Methodology/Principal Findings:**

We addressed this issue based on a Ca^2+^ indicator dye (fluo-4) and a protein Ca^2+^ sensor (yellow cameleon). Using fluo-4 AM dye, we found spontaneous Ca^2+^ spikes in 18% of rat SCN cells in acute brain slices, but the Ca^2+^ spiking frequencies showed no day/night variation. We repeated the same experiments with rat (and mouse) SCN slice cultures that expressed yellow cameleon genes for a number of different circadian phases and, surprisingly, spontaneous Ca^2+^ spike was barely observed (<3%). When fluo-4 AM or BAPTA-AM was loaded in addition to the cameleon-expressing SCN cultures, however, the number of cells exhibiting Ca^2+^ spikes was increased to 13∼14%.

**Conclusions/Significance:**

Despite our extensive set of experiments, no evidence of a circadian rhythm was found in the spontaneous Ca^2+^ spiking activity of SCN. Furthermore, our study strongly suggests that the spontaneous Ca^2+^ spiking activity is caused by the Ca^2+^ chelating effect of the BAPTA-based fluo-4 dye. Therefore, this induced activity seems irrelevant to the intrinsic circadian rhythm of [Ca^2+^]_c_ in SCN neurons. The problems with BAPTA based dyes are widely known and our study provides a clear case for concern, in particular, for SCN Ca^2+^ spikes. On the other hand, our study neither invalidates the use of these dyes as a whole, nor undermines the potential role of SCN Ca^2+^ spikes in the function of SCN.

## Introduction

The circadian clock system governs various daily rhythms in physiological activities, from cellular autonomic activities to animal behaviors. The key component of this system is the suprachiasmatic nucleus (SCN) [1]–[3]. The SCN is composed of a pair of nuclei, each of which is comprised of 8,000 neurons approximately [4], [5]. One key property of SCN neurons is the circadian oscillation in their spontaneous action potential (AP) firing rates [6]–[9]. Recent studies have shown that intracellular circadian oscillations are the result of transcription-translation feedback loops arising from multiple “clock genes” [1], [10]. Yet, no one has shown how clock gene products modulate AP firing frequencies in SCN neurons.

The concentration of cytosolic free Ca^2+^ ions ([Ca^2+^]_c_) is a general intracellular messenger regulating a variety of different cellular processes, including membrane potentials and gene expression. Thus, it is possible that [Ca^2+^]_c_ is a mediator coupling clock gene oscillations and AP firing rhythms in SCN neurons [9], [11]–[14]. Indeed, Colwell observed that the level of [Ca^2+^]c was higher during day time than night time by using fura-2 [15]. Subsequently, a few years ago, one of us observed circadian rhythms in [Ca^2+^]_c_ of cultured mouse SCN neurons that expressed yellow cameleon, a Ca^2+^-sensing protein [14], [16], [17]. In the presence of tetrodotoxin, the AP firing activity of SCN disappeared completely but the circadian rhythm of [Ca^2+^]_c_ was persistent. On the other hand, the amplitudes of circadian oscillations, both in AP firing rate and [Ca^2+^]_c_, were significantly reduced by ryanodine and 8-bromo-cADP ribose. Thus, the release of Ca^2+^ from internal Ca^2+^ stores might have driven AP circadian oscillations in SCN neurons.

In addition to the slow circadian modulation of [Ca^2+^]_c_, there have been reports on Ca^2+^ spikes that have a duration in the order of several seconds to a minute in SCN neurons. For example, van den Pol et al. discussed SCN Ca^2+^ spikes in connection with neuro-glia interactions [18], [19]. Their studies were based on fluo-3 and fura-2 imaging of cultured SCN cells. More recently, using patch pipettes filled with fura-2, Irwin and Allen demonstrated that electrical stimulation of retinohypothalamic tracts could evoke Ca^2+^ spikes [20]. They were strongly correlated with AP firing in SCN neurons. Also, Ikeda et al. observed spontaneous Ca^2+^ spikes in fura-2 stained acute SCN slices [21].

Knowing that SCN cells support two rather temporally distinct [Ca^2+^]_c_ dynamics, we investigated their relationship: In particular, we looked for the existence of circadian modulation in the Ca^2+^ spiking frequency. We also investigated the nature of spontaneous Ca^2+^ spikes in SCN based on two different Ca^2+^ indicators, fluo-4 dye and yellow cameleon.

## Materials and Methods

### Ethics Statement

All experimental procedures and protocols above were in accordance with the guidelines established by the Committee of Animal Research Policy of Korea University College of Medicine and Toyama University.

### Acute Brain Slice Preparation

We used standard procedures to prepare acute brain slices containing SCN. More specifically, we used male Sprague-Dawley (SD) rats of 12–15 days old that had been bred under a 12∶12 h light-dark cycle (lights on from 0800-2000) at a constant temperature (22∼24°C). For the night time recordings, the animals were maintained in a reversed light-dark cycle (lights on from 2000-0800) for at least 2 weeks before the slice preparations. Once the rats had been deeply anesthetized with sodium pentobarbital (100 mg/kg body weight, i.p.), we prepared coronal hypothalamic slices (300 µm) containing the SCN with a vibratome (TPI, St. Louis, MO, USA) in ice-cold low-Ca^2+^ artificial cerebrospinal fluid (ACSF) containing (in mM) 124 NaCl, 4.0 MgSO_4_, 3 KCl, 1.25 NaH_2_PO_4_, 26 NaHCO_3_, 0.5 CaCl_2_, and 10 glucose, being adjusted to 300 mosmol/kg with sucrose and saturated with 5% CO_2_ and 95% O_2_. Typically, two or three brain slices were cut per each preparation but only the slice containing the rostrocaudal center of the SCN was used for recording. Therefore, each brain slice represents one animal. For the nighttime recordings, the brain slices were prepared between zeitgeber time (ZT) 11 and ZT12 (i.e., within an hour before lights off). For both daytime and nighttime recordings, the brain slices were incubated for at least 1 hour in a chamber containing oxygenated low-Ca^2+^ ACSF (1.3 mM MgSO_4_, 1 mM CaCl_2_).

### Organotypic Slice Cultures

The organotypic SCN slice cultures were made from 2- to 3-day-old SD rats or from C57BL/6J mice as described previously [16]. The brains were quickly excised and dropped into ice-cold, filter-sterilized, low-Ca^2+^ ACSF. Coronal hypothalamic slices containing the SCN were cut at 400 µm for rats or 350 µm for mice using a vibroslicer (World Precision Instruments, Sarasota, FL, USA) on a clean bench. We cut two or three sequential slices from the rostral to the caudal brain starting at the rostral end of the anterior commissure. These slices were transferred to a 0.40-µm filter cup (Millicell-CM, Millipore, Bedford, MA, USA), placed in a standard 6-well plate, and cultured with 1 mL of medium that consisted of 50% Eagle's basal medium, 25% Earle's balanced salt solution, and 25% heat-inactivated horse serum, supplemented with 5 mg/mL glucose and 1∶100 Glutamax (Invitrogen, Carlsbad, CA, USA). The cultures were maintained in a CO_2_ incubator at 36°C and 5% CO_2_. The culture medium was changed every 3–4 days. Only the slice culture that clearly shows the rostrocaudal center of the SCN was used for imaging.

### Patch-Clamp Recording

After preparation, each acute SCN slice was transferred to a recording chamber and superfused continuously with warmed regular ACSF (1.3 mM MgSO_4_, 2 mM CaCl_2_, 25–30°C, pH 7.4) supplemented with gentamicin (50 mg/L) at a flow rate of 0.5 mL/min. The ACSF was continuously bubbled with 95% O_2_/5% CO_2_. The whole-cell patch recordings were taken from 1 to 6 h after the slice preparation. The patch micro-electrode (∼2 µm tip diameter; ∼5 MΩ resistance) was filled with a solution containing (in mM) 140 K-gluconate, 10 HEPES, 2 MgCl_2_, 1 CaCl_2_, 11 EGTA, 2 K_2_ATP; (pH 7.3; osmolality 285–290 mosmol/kg) in accordance with methods described elsewhere [22]. The cell approach and the sealing were guided by series resistance changes monitored with current responses to the standard square voltage pulse. After successful contact had been made between the micro-electrode and the target neuron, we applied a negative pressure to form a giga seal (2–5 GΩ). An additional gentle suction was used to disrupt the membrane and produce a whole-cell mode.

The membrane potential was measured continuously over 20 min in a current-clamp configuration using an Axoclamp 700A amplifier, a Digidata 1200 interface, and the pCLAMP 9.0 data acquisition/analysis software (Axon Instruments, Union City, CA, USA). The sampling rate was 10 kHz. The action potential peaks were detected by pCLAMP 9 data acquisition/analysis software, and the digitized data were analyzed Origin™ ver.8.0 (OriginLab Co., Nortnampton, MA, USA) with custom-made programs.

### Measurement of Intracellular Ca^2+^ Using Fluo-4

We used two different methods for fluo-4 dye loading into SCN neurons. First, we transferred acute SCN slices into a small home-built staining chamber and incubated them for 1 hr in oxygenated regular ACSF containing 20-µM fluo-4 acetoxymethyl ester (fluo-4 AM; Invitrogen), following the methods of Ikegaya et al. [23]. The stained slice was then transferred to a small recording chamber and washed for at least 20 min by perfusion of oxygenated regular ACSF in preparation for the subsequent imaging experiment. This technique was used for the recording of multiple [Ca^2+^]_c_ responses from multiple cells in a single slice. We used the second method to make simultaneous measurements of [Ca^2+^]_c_ concentrations and membrane potentials in single SCN neurons, also by loading fluo-4 through patch micro-electrodes. The patch micro-electrodes were back-filled with an internal solution containing (in mM) 132 K-gluconate, 8 KCl, 8 NaCl, 10 HEPES, 0.5 MgCl_2_, 4 Mg-ATP, and 0.4 Na-GTP (pH 7.3; osmolality 285–290 mosmol/kg) and filled with an internal solution containing fluo-4 pentapotassium salt (30–180 µM; Invitrogen) according to the methods described by Eilers and Konnerth [24]. Whole-cell current clamp recordings were taken as described above, and the SCN neurons were viewed with a laser-scanning confocal system (Fluoview 500, Olympus, Tokyo, Japan) attached to an upright microscope (BX51WI, Olympus) with a water immersion objective lens (Olympus UMPlanF1/10×/0.30W or LUMPlanF1 40×/0.8W). An argon laser (488 nm) was used for the excitation, and a green emission filter (515±10 nm) was used to observe the fluorescent images. The series of image data was acquired and recorded by the Fluoview™ software package (Olympus). The fluorescent emission images were collected every 1.12 sec for a period of at least 18 minutes. Each image had a spatial resolution of 512×512 pixels. The relative fluorescent intensity change (ΔF = F- F_0,_ where F_0_ is the initial fluorescent intensity at the cell body of the SCN neuron) was calculated in order to monitor [Ca^2+^]_c_ variations.

### Measurement of Intracellular Ca^2+^ Using Yellow Cameleon

Yellow cameleon genes (YC3.6 [25]) linked to a neuron-specific enolase promoter (pNSE/YC) or to a cytomegalovirus promoter (pCMV/YC) were transfected into rat or mouse SCN cultures using a Helios Gene Gun system (Bio-Rad Laboratories, Hercules, CA, USA), as described in earlier work [16], [17]. The technique involves coating small gold particles (0.6 µm, 5 mg) with vector-carrying YC genes (20 µg) according to the manufacturer's instructions and then blasting them into the slice cultures at days 7 or 8 *in vitro*.

We used two different imaging setups for the cameleon imaging. The primary setup was designed for Ca^2+^ imaging analysis at a high sampling rate. In this setup, images were acquired by an iXon EMCCD camera (Andor, Belfast, Northern Ireland, UK) mounted on an inverted microscope (IX71, Olympus) that was equipped with a 10× objective lens (LUCPlanFL, NA0.30, Olympus), a mercury arc lamp, and a Dual-view optical module (Optical-insights, Tucson, AZ, USA). For excitation we used a filter set (OI-05-Ex, Chroma Technology, Rockingham, VT, USA) that included an excitation bandpass filter (436±20 nm) and a dichroic mirror (455DCLP, Chroma). In order to minimize the photo-bleaching, two neutral-density filters (ND6, ND50, Olympus) were placed in the excitation light path as well. The fluorescent emission was separated by a dichroic mirror (505DCXR, Chroma) and two emission bandpass filters (480±30 nm and 535±40 nm) installed in the Dual-view module. An electromagnetic shutter (Vincent Associates, Rochester, NY, USA) was placed in front of the lamp house. The shutter control, image acquisition, and online image processing were coordinated by software based on the Andor SDK subroutine packages. A SCN slice that had successful YC expression was transferred to the recording chamber and superfused with warmed oxygenated regular ACSF. The fluorescent emission images were collected every 1.5 seconds for more than 5 minutes. The acquired images were further analyzed by ImageJ (NIH, Bethesda, Maryland, USA) with custom-built software.

The second imaging setup was based on the original cameleon imaging system created for the long-term monitoring of [Ca^2+^]_c_ levels in SCN neurons [16], [17]. That setup uses an inverted fluorescent microscope (Axiovert 405M, Carl Zeiss) equipped with a custom-built microscope stage CO_2_ incubator, a mercury arc lamp, an excitation filter (435.8 nm DF10, Omega Optical, Brattleboro, VT, USA), an excitation neutral density filter (ND.5, Omega Optical), a dichroic mirror (455DRLP, Omega Optical), and a 20× objective lens (Plan-Neofluar 20×, NA0.5, Carl Zeiss). The two emission bandpass filters (480DF30 and 535DF25, Omega Optical) are switched by a filter changer wheel (C4312, Hamamatsu Photonics, Hamamatsu, Japan). We acquired the resulting image pairs through a cooled CCD camera (C6790, Hamamatsu) at a sampling rate of 1 frame per 3 seconds for detecting Ca^2+^ spikes or of 1 frame per 10 minutes for detecting circadian [Ca^2+^]_c_ oscillations. Argus-HiSCA imaging software (Hamamatsu) was used to control an electromagnetic shutter (Copal, Tokyo, Japan), a filter changer wheel, and image acquisition.

### Statistical Analyses

Means were calculated along with standard errors. Single pairwise comparisons were analyzed with the two-tailed Student's t-test. A one-way analysis of variance (ANOVA) was used to compare the population percentage of differently prepared SCN slices and the different Ca^2+^ spiking responses to different levels of fluo-4 AM concentration.

## Results

### Spontaneous [Ca^2+^]_c_ Spiking Activities in Fluo-4 AM Loaded SCN Slices

**Figure 1:**
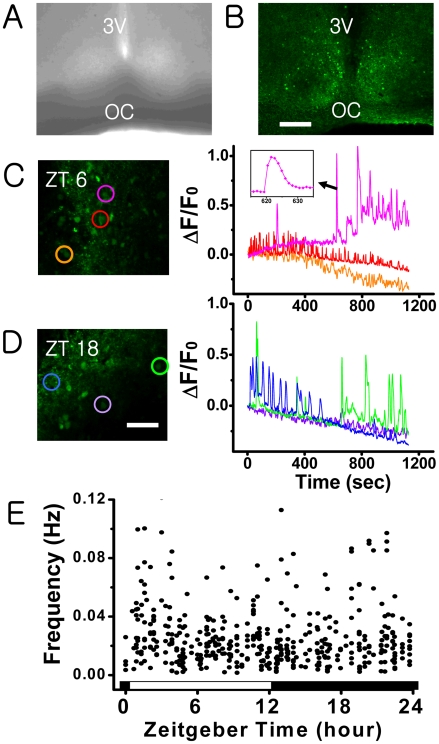
Spontaneous Ca^2+^ spikes in rat acute SCN slices: Phase contrast image (A) and fluo-4 AM (20 µM) stained fluorescent image (B) of a hypothalamic brain slice containing rat SCN (scale bar, 200 µm). (C and D) Time series of spontaneous [Ca^2+^]_c_ spiking activities of SCN neurons obtained at ZT 6 and ZT 18 (scale bar, 50 µm). Each colored circular mark in C and D matches with the time series shown in the same color. The bandwidth of each calcium spike varies significantly from one cell to the other, typically ranging from 5–60 seconds (cf. graph in inset). (E) The mean frequency of spontaneous Ca^2+^ spikes in SCN as a function of zeitgeber time. Each dot marks the mean frequency of the Ca^2+^ spikes exhibited by one cell during a period of 800–1000 sec. The data were collected from the [Ca^2+^]_c_ active cells in 1–3 different slices each hour, totaling 475 cells.

We prepared and stained the acute slices of SCN with a bath application of 20 µM fluo-4 AM (Fig. 1A and B) as described in Materials and Methods, and used a laser-scanning confocal microscope to monitor their short-term [Ca^2+^]_c_ activities over a duration of 800 seconds. Time traces exhibiting Ca^2+^ spikes are shown in Fig. 1C and D. Typically, the traces showed neither a discernable temporal pattern nor cell-specific characteristics: the width of each spike and the inter-spike interval both varied significantly in time and from one cell to the other (Fig. 1C and D). The observation time was limited due to dye bleaching. Therefore, we monitored a total of 2639 stained cells in 82 slices during various circadian phases and found that 18±1% (mean±SEM) of them exhibited spontaneous Ca^2+^ spikes. Collectively, our extensive set of data showed no indication of circadian variation. In other words, unlike the AP firing frequency of SCN neurons, the Ca^2+^ spiking frequency showed no circadian variation (Fig. 1E).

Right after the [Ca^2+^]_c_ imaging experiments were done, some slices were double-stained with an astrocyte maker, sulforhodamine 101 to determine if the active cells producing Ca^2+^ spikes were a neuron or glia [23], [26]. The majority of the active cells turned out to be neurons (76±5%, 81 out of 106 cells in 9 slices).

### Absence of Sspontaneous Ca^2+^ Spikes in Cameleon-Transfected SCN Slices

**Figure 2:**
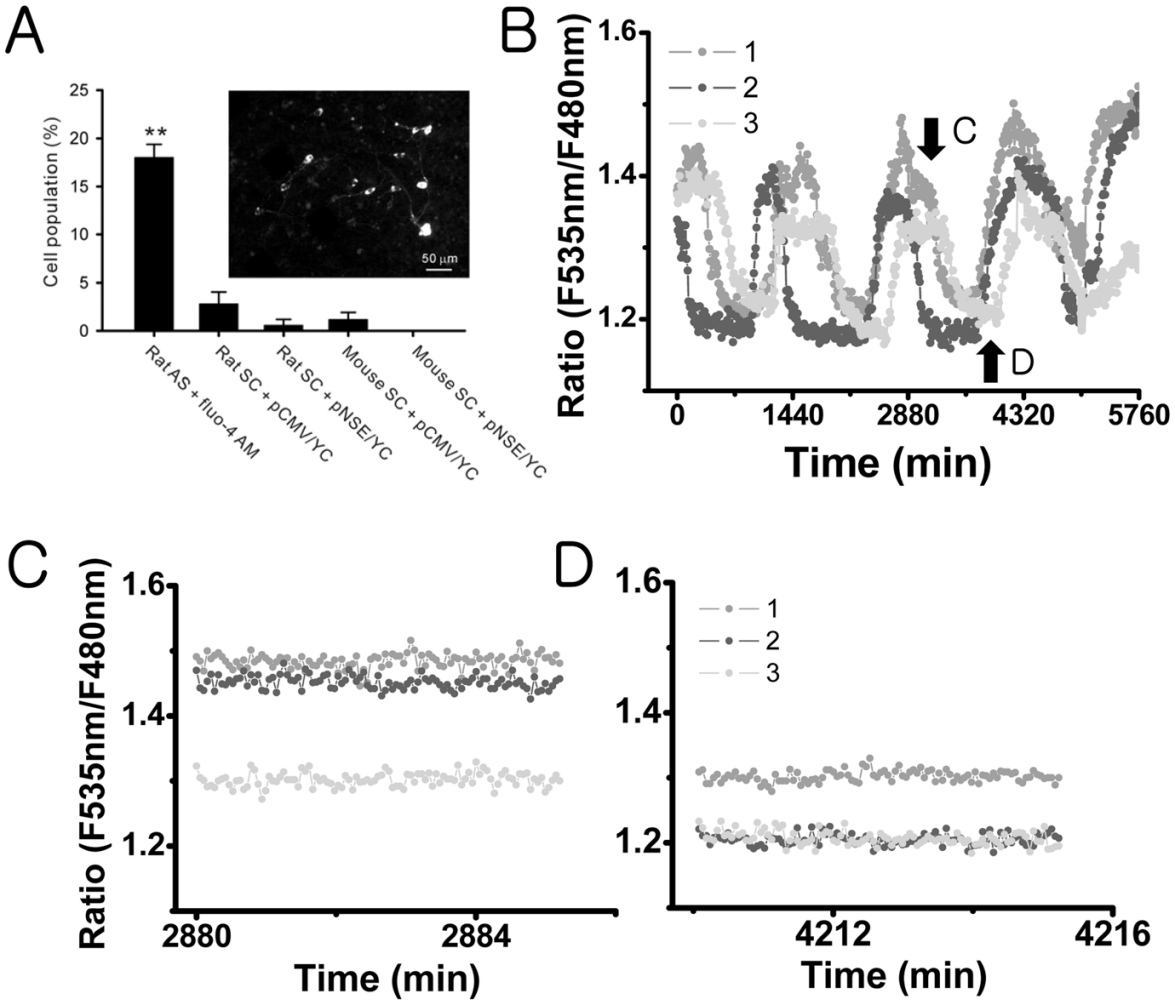
None or fewer Ca^2+^ spiking activities in cultured SCN cells that express yellow cameleon: (A) Comparison of cell populations that displayed spontaneous Ca^2+^ spikes in different SCN preparations. The fluo-4 AM-loaded rat acute slice (rat AS + fluo-4 AM) contains Ca^2+^ spiking cells in 18% of the total cells (as in Fig. 1). This ratio was significantly larger than that in rat slice cultures (rat SC) or in mouse slice cultures (mouse SC). **P<.01 by one-way ANOVA. The slice cultures that express yellow cameleon with CMV promoter (+pCMV/YC) had slightly larger number of Ca^2+^ spiking cells in comparison with those that express yellow cameleon with NSE promoter (+pNSE/YC), but the difference was not statistically significant. An example fluorescent image of mouse SC + pNSE/YC is shown on the left (scale bar, 50 µm). (B) Long-term traces (sampling rate at 1 frame per 10 min) of the level of [Ca^2+^]_c_ in three different SCN neurons in the mouse SC + pNSE/YC represent robust circadian oscillations. The imaging done at the higher sampling rate (1 frame per 3 seconds) was taken at the approximate plateau (C) and trough (D) of the circadian [Ca^2+^]_c_ rhythms, with no [Ca^2+^]_c_ spiking activities being detected in these SCN neurons.

We also looked for the occurrence of spontaneous Ca^2+^ spikes in rat and mouse slice cultures transfected with pCMV/YC or pNSE/YC. In the rat slice cultures, Ca^2+^ spikes were observed only in 2.8±1.2% of the cells (number of slices = 5) transfected with pCMV/YC and in 0.6±0.6% of the cells (number of slices = 5) transfected with pNSE/YC. In the mouse slice cultures, Ca^2+^ spikes were observed only in 1.2±0.7% of the cells (number of slices = 5) transfected with pCMV/YC and were not observed at all in cells transfected with pNSE/YC (number of slices = 5). These percentages were much smaller than those for the acute rat SCN slices that were stained with fluo-4 AM (F_4,97_ = 8.5, *P*<.01 by one way ANOVA; Fig. 2A).

Using mouse slice cultures transfected with pNSE/YC, we confirmed the presence of circadian rhythms in [Ca^2+^]_c_ in 62.8±3.8% of the SCN neuronal population (27 out of 44 neurons; number of slices = 6; Fig. 2B). After monitoring the circadian [Ca^2+^]_c_ rhythms for several cycles, we imaged the SCN neurons at a much higher sampling rate (1 frame per every 3 seconds) for 5 minutes during different circadian phases (Fig. 2C and D). There was no Ca^2+^ spiking activity even at the plateau phase.

### Inducible Ca^2+^ Spikes in Cameleon-Expressed SCN Cells

**Figure 3:**
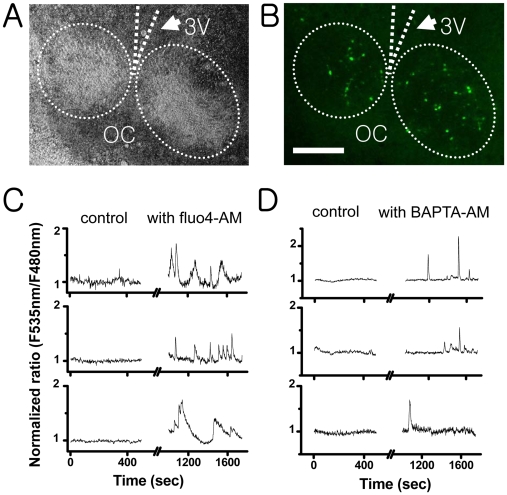
The effect of fluo-4 AM and BAPTA AM on the [Ca^2+^]_c_ level of rat SCN cells expressing cameleon (pCMV/YC): The transmitted-light image (A) and fluorescent-light image (B) of a hypothalamic slice culture showing a pair of SCN (scale bar, 200 µm). This particular slice culture contained approximately 70–90 cameleon-expressing SCN cells. The cameleon-expressing cells were recorded for 450 seconds under the circulation of oxygen-saturated ACSF. Then the same samples were incubated under the ACSF containing 2.5-µM fluo-4 AM (C) or BAPTA AM (D) for 15 minutes, washed, and recorded again for another 500 seconds. None of the cells expressing cameleon had spontaneous Ca^2+^ spikes before the treatment with fluo-4 AM or BAPTA AM, but approximately 13∼15% (17 out of 118 cameleon-expressed cells in a fluo-4 AM-treated sample, 20 out of 155 cells in a BAPTA AM-treated sample) of the cameleon-expressing cells exhibited Ca^2+^ spikes after the treatment with fluo-4 AM or BAPTA AM. (C) and (D) are a typical example of the responding cells.

To understand the lack of Ca^2+^ spikes in cameleon-transfected SCN cells, we examined the effect of additional fluo-4 AM loading on the cameleon-transfected SCN cells (Fig. 3A and B). At a concentration of 2.5 µM, fluo-4 AM evoked Ca^2+^ spikes (Fig. 3C) in 14% of the SCN cells examined (17 out of 118 cameleon-expressed cells in 4 slices). Similarly, the treatment with BAPTA-AM (2.5 µM) could also induce Ca^2+^ spikes in 13% of the SCN cells (20 out of 155 cells in 5 slices) (Fig. 3D). Since both were dissolved in DMSO, we also performed several DMSO control experiments to find no Ca^2+^ spikes (9 slices).

### The Modulations in the Level of [Ca^2+^]_c_ and the AP Firing Pattern of SCN Neurons by Fluo-4 Loading

**Figure 4:**
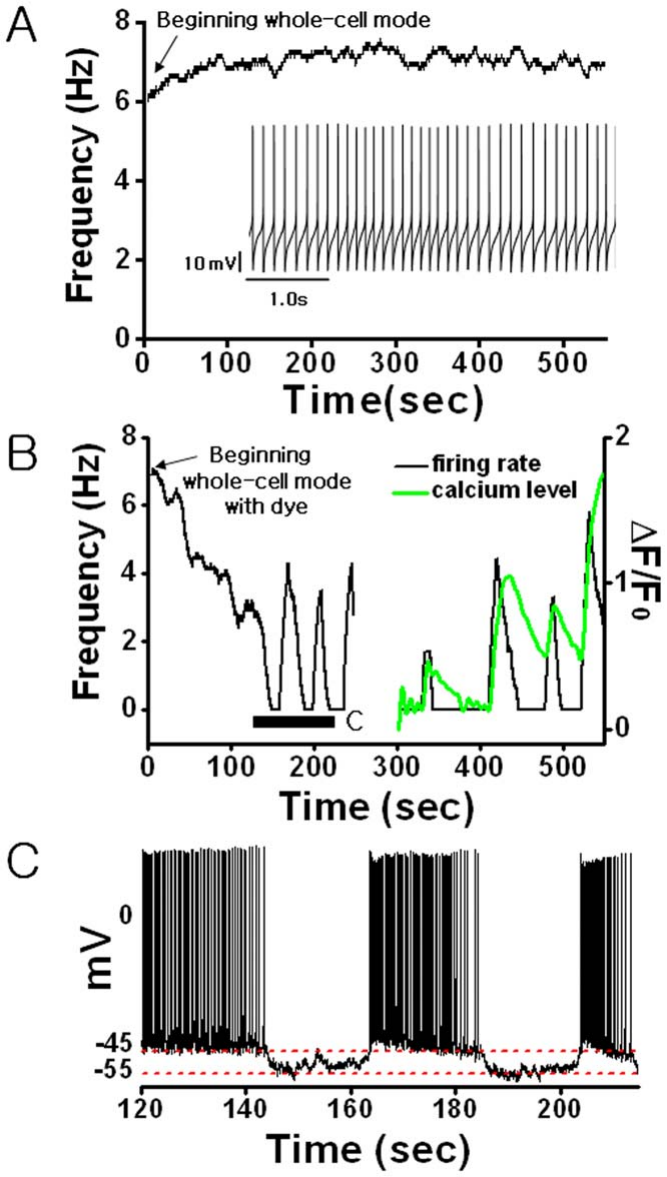
The loading effect of fluo-4 Ca^2+^indicator dye on the AP firing activity of a SCN neuron: (A) A typical spontaneous firing pattern and the time trace of mean firing rate of a SCN neuron in an acute rat hypothalamic slice. (B) The time trace of mean firing rate (black) and the level of [Ca^2+^]_c_ (green) of a SCN neuron following the fluo-4 AM dye injection (100 µM). The mean firing rate is computed based on a 10-second sliding window. The time “0” denotes the onset of the whole-cell mode. (C) Time series of AP spiking activities during the fluo-4 AM dye loading as marked in B. The red dashed lines guide the resting membrane potential.

We made fluo-4 imaging and current clamp recording simultaneously using fluo-4 dye loaded micro-pipettes in order to see the direct effect of fluo-4 calcium dye on the level of [Ca^2+^]_c_ and the AP firing pattern in individual SCN neurons. Figure 4A shows a typical pattern of spontaneous AP firing in a SCN neuron in the absence of fluo-4 dye. It is quite regular and shows a stable resting membrane potential (*Vm*). In many cases, this regular pattern changed significantly with an addition of fluo-4 (pipette concentration in the range of 80–120 µM). As the dye diffused from the patch pipette into the target neuron, during the first 100 seconds or so, the AP firing rate gradually decreased from 7 Hz to 3 Hz, and at around 150 seconds the AP activity disappeared rather quickly (Fig. 4B). This change was accompanied by a decrease in *Vm* from -45.0±1.6 mV to -54.0±4.0 mV (Fig. 4C). Subsequently, the AP firing rate showed a large change, producing a random sequence of sharp peaks with each peak matched with a Ca^2+^ spike in one-to-one manner (Fig. 4B). During each peak, the value of *Vm* recovered more or less to the initial level (Fig. 4C).

Regarding the Ca^2+^ spikes in the SCN cells induced by fluo-4 dye, the following facts were further confirmed. First, the results were concentration-dependent. When the pipette dye concentration was below 60 µM, no Ca^2+^ spikes were apparent, and there was no significant change in either the AP firing rate or *Vm* (10 neurons in 6 slices; Table 1). Conversely, when the dye concentration was more than 150 µM, *Vm* and [Ca^2+^]_c_ increased steadily until the SCN neurons died (8 neurons in 4 slices). When the dye concentration was in the intermediate range of 80–120 µM, about half (9 out of 18 cells in 8 different slices) of the tested SCN neurons exhibited Ca^2+^ spikes, and they showed a decrease in *Vm* of 6.7% (*P*<.05 by Student's *t*-test).

**Table 1:** The change of the resting membrane potential of SCN neurons induced by fluo-4 dye loading.

fluo-4 concentration	>150 µM	80∼120 µM	<60 µM
**Number of neurons**	n = 8/8	n = 9/18	n = 10/10
**Initial ** ***Vm (mV)***	−43.7±1.6	−43.4±0.8	−43.2±2.1
**Steady state ** ***Vm (mV)***	Unstable	−46.3±1.3*	−41.7±2.0

‘Initial’ and ‘steady state’ refer to the first 30 seconds of dye loading and the time just before the emergence of the first Ca^2+^ spike, respectively. The given values represent mean±SEM.

*p<0.05, t-test.

### Fluo-4 AM Concentration Dependence of Ca^2+^ Spikes

**Figure 5:**
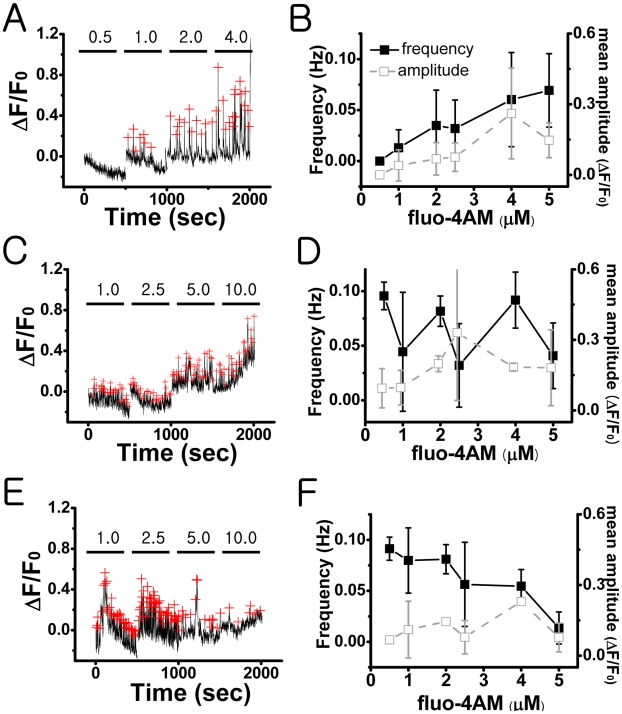
Heterogeneous response of SCN cells to the increasing concentration of fluo-4 AM in rat slice cultures of SCN: The dependency of spontaneous [Ca^2+^]_c_ spiking activity on the concentration (0.5–10 µM) of fluo-4 AM was examined. The mean frequency of calcium spikes was gradually increased in about 49% (A–B) of the active populations, had no systematic change in 35% (C–D), and decreased in 16% (E–F). The red crosses mark the peak positions. All error bars represent a standard error. The data were collected from a total of 76 active cells in 3 different slices. A, C, and E are three representative cases corresponding to the three different subpopulations.

The frequency of induced Ca^2+^ spikes was not a simple function of fluo-4 AM concentration. We observed quite heterogeneous responses following a stepwise increase of fluo-4 AM concentration (0.5–10 µM; Fig. 5). Based on these responses, SCN cell populations could be divided broadly into three different groups. For the majority of the cells (48.7%, 37 out of 76 cells in 3 slices), the amplitude as well as the frequency of Ca^2+^ spikes were an increasing function of fluo-4 AM concentration (Fig. 5A and B; Frequency: F_5,105_ = 14.6, Amplitude: F_5,105_ = 3.8, *P*<.05). For the next majority of the cells (35.5%, 27 out of 76 cells in 3 slices), there was no systematic trend (Fig. 5C and D) both in the frequency and the amplitude. Finally, for the rest of the cell population (15.8%, 12 out of 76 cells in 3 slices), the frequency was a decreasing function of fluo-4 AM concentration (Fig. 5E and F; F_5,33_ = 7.74, *P*<.05), while the amplitude could be decreasing, unchanging, or increasing, depending on the individual cells. In other words, the systematic trend in the frequency did not guarantee the same trend in the amplitude.

## Discussion

### The Rate of “Spontaneous Ca^2+^ Spikes” Showed No Circadian Variation

The spontaneous AP firing rates and the cytosolic concentration of free calcium ions [Ca^2+^]_c_ of SCN neurons are known to have circadian rhythms. Also reported was the existence of [Ca^2+^]_c_ transients in SCN having a typical duration of tens of seconds. Here, we termed these Ca^2+^ transients as “Ca^2+^ spikes.” One immediate question following these observations would be if the Ca^2+^ spiking activity of SCN neurons can exhibit any circadian variation similar to the circadian AP firing activity, and here we studied that issue. The level of [Ca^2+^]_c_ was visualized by Ca^2+^ sensitive fluorescent protein cameleon as well as fluo-4 AM dye. Many fluo-4-loaded SCN cells exhibited Ca^2+^ spikes spontaneously, but our extensive set of fluo-4 imaging experiments based on 82 acute SCN slices found no day/night variation in the Ca^2+^ spiking rates (Fig. 1E): the Ca^2+^ spiking activities of SCN cells during the night time were as strong (or weak) as those of day time, or vice versa. In our earlier studies that investigated the progressive changes in [Ca^2+^]_c_ for the duration longer than a circadian cycle, we used organotypic cultures of SCN slices that were transfected by pNSE/YC genes [14], [16], [17]. The level of [Ca^2+^]_c_ was estimated with YC2.1 at every 10 minute in order to minimize photo-bleaching: with this slow image acquisition rate, the presence or the absence of Ca^2+^ spikes could not be determined [16]. In the present study, we used YC3.6 (either pNSE/YC or pCMV/YC), and [Ca^2+^]_c_ images were acquired at every 3 seconds to monitor Ca^2+^ spiking events. The cameleon YC3.6 is a newer version of YC2.1 giving a better spatiotemporal resolution [25]. We observed that YC3.6-transfected organotypic cultures of SCN could exhibit well-defined circadian rhythms in the level of [Ca^2+^]_c_, just as in the YC2.1-transfected cultures. However, in the same preparations almost no Ca^2+^ spike was observed even at the peak of [Ca^2+^]_c_ circadian oscillation (Fig. 2). This was definitely not due to the lack of sensitivity of YC3.6 because spontaneous Ca^2+^ spikes were observed in other hyperthalamic neurons neighboring SCN (Figure S1).

### “Spontaneous Ca^2+^ Spikes” Were Induced by Fluo-4 Ca^2+^ Dye Loading

Our study found Ca^2+^ spikes to be present in about 18% of fluo-4-loaded SCN cells, while Ca^2+^ spikes were observed only in 1–3% of cultured SCN cells, which expressed pCMV/YC genes, and in 0–0.6% of cultured SCN neurons, which expressed pNSE/YC genes. In addition, the population of cultured SCN cells that displayed Ca^2+^ spikes was significantly increased to about 15% by an additional loading of fluo-4 AM or BAPTA-AM (Fig. 3). Taken all together, we could conclude that the observed spontaneous SCN Ca^2+^ spikes were not intrinsic to SCN but induced by fluo-4 dye loading.

In order to verify the effect of calcium dye loading on the level of [Ca^2+^]_c_ and the spontaneous AP firing activity in SCN neuron, more directly, we performed a patch-clamp recording along with fluo-4 imaging. Most notably, the loading of fluo-4 through the patching micro-pipette to the cell initially decreased both the AP firing rate and the level of resting membrane potential (Fig. 4) of SCN neurons. The decreases were not due to the whole-cell patching itself, as no such decrease was observed in our control experiments. Soon after the spontaneous AP firing activity of SCN neurons disappeared completely, an intermittent sequence of AP firings in groups, each of which well matched to a Ca^2+^ spike in one-to-one manner, emerged (Fig. 4B). The bursting mode of AP firings is quite unusual for SCN neurons, since their spontaneous AP firing pattern is typically regular as shown in Fig. 4A.

It has been proposed that Ca^2+^ influx via L-type Ca^2+^ channels [9] or BK type calcium-activated potassium channel activities [27] act to regulate spontaneous AP firing frequencies in SCN neurons. On the other hand, Aguilar-Roblero et al. [28] have demonstrated that AP firing frequencies in SCN neurons can be increased by ryanodine receptor activators (e.g., caffeine) and decreased by ryanodine receptor blockers (e.g., dantrolene): in other words, the mobilization of Ca^2+^ store by ryanodine-sensitive intracellular Ca^2+^ channels can modulate AP firing rate. In any case, all of these studies indicate a close connection between the level of [Ca^2+^]_c_ and the AP firing activity of SCN neurons, and thus it is not too surprising that in our whole-cell patching experiment each Ca^2+^ spike matches with a group of bursting AP spikes in one-to-one manner.

Although our current investigation did not answer how exactly the chelation of Ca^2+^ by dye loading interfered with the cell's intrinsic calcium kinetics to produce Ca^2+^ spikes, there is no doubt that they were induced by fluo-4 dye loading. The observed Ca^2+^ spikes could be a natural response of SCN neurons trying to recover their original cytosolic [Ca^2+^]_c_ levels. Some mechanisms were discussed earlier for the cause of SCN Ca^2+^ spikes. For example, van den Pol et al. [18] and Obrietan and van den Pol [19] found that they could be induced by the modulation of neurotransmitter, such as gamma-aminobutyric acid (GABA) and glutamate. Also, more recently, Irwin and Allen [20] demonstrated that the Ca^2+^ influx through L-type Ca^2+^ channels following the electrical stimulation of retinohypothalamic tracts could induce Ca^2+^ spikes in SCN neurons. The Ca^2+^ spikes observed in our experiments were neither induced by neurotransmitter modulations nor electrical stimulations. Yet, they were visually very similar to the ones reported earlier [18]–[20]. In any case, the SCN Ca^2+^ spikes all were not an intrinsic spontaneous activity but induced.

### Differences between Cameleon and Fluo-4 Calcium Dye

It has been known that BAPTA-based high affinity calcium dyes, such as fura-2, fluo-3, and fluo-4 can have a significant intracellular Ca^2+^ buffering and induce some non-intrinsic phenomena. For example, Ca^2+^ waves supported by the network of astrocytes can be completely blocked by the chelating action of fura-2 [29]. They can also delay the calcium signals initiated by extracellular calcium influx or release of calcium from intracellular stores by 5- to 20-fold [30]. Therefore, it is not too surprising that Ca^2+^ spikes in SCN neurons have appeared as an artifact of the Ca^2+^ chelation of fluo-4 dye.

When the chelation by indicator dye becomes an issue, its affinity (the inverse of dissociation constant K_d_) and concentration are two important factors to be considered. High-affinity chelators trap calcium efficiently, and the presence of them at high concentration can sequester intracellular free Ca^2+^ significantly. We believe that the affinity (K_d_ = 345 nM) and the concentration (5∼20 µM) of fluo-4 were high enough to interfere with the intrinsic calcium kinetics of SCN neurons and resulted in non-intrinsic Ca^2+^ spikes. The affinity of cameleon YC3.6 (K_d_ = 250 nM) is 1.73 times higher than that of fluo-4 (K_d_ = 345 nM). Therefore, we suspect that the cytosolic concentrations of cameleon protein expressed within the SCN neurons were quite lower than that of fluo-4 dye that we used. As for the concentration, another important factor to be considered is the stability of the dye. It is well known that cameleon is far more stable than any BAPTA-based calcium indicators with respect to dye leakage, exhaustion, or cumulative photodestruction [31]. Therefore, the effective concentration of cameleon inside the cells could have been much lower than that of the fluo-4 dye.

The concerns with BAPTA based dyes are well known and our extensive set of data provides a clear case for concern, especially, regarding SCN calcium dynamics. However, our result does not invalidate the use of these dyes.

### SCN Cells Are Heterogeneous with Regard to Their Cytosolic Ca^2+^ Buffering Systems

Previous research has shown that Ca^2+^ buffering molecules, such as calbindin D-28k (K_d_ is about 300 nM), may play a critical role for the function of SCN neurons [32], [33]. The type of Ca^2+^ buffering proteins and their localization within the SCN may differ depending on the particular animal species [34], [35], the developmental stages [36], and the circadian timings [37]. For example, Ikeda et al. (2003a) has shown that the nucleus and the cytoplasm of SCN neurons can support quite different Ca^2+^ kinetics at a given time.

Several earlier studies found BAPTA-based fluorescent Ca^2+^ dyes often compete with the Ca^2+^ buffering system of involved cells. In fact, the Ca^2+^ chelating effects of fura-2 could be used to estimate the capacity of the Ca^2+^ buffering systems in various types of cells [38]–[40]. Accordingly, we suspect that the heterogeneous effect of fluo-4 AM on the generation of Ca^2+^ spikes (Fig. 5 and Table 1) may reflect the different Ca^2+^ buffering capacities of SCN cells. The SCN neurons have been categorized into several different subgroups [41], [42], and it is quite possible that the heterogeneity of the Ca^2+^ buffering systems may underlie the existence of these subgroups.

### Concluding Remarks

Our experimental studies demonstrated that Ca^2+^ spikes, having a typical bandwidth of tens of seconds, could be induced in SCN neurons by the Ca^2+^ chelating effect of BAPTA-based dyes. The firing rate of these induced SCN Ca^2+^ spikes did not show a circadian variation. Furthermore, Ca^2+^ spikes were very rarely observed when a reporter protein was used instead. Based on these facts, we conclude that spontaneous Ca^2+^ spikes in the presence of BAPTA-based dyes are most likely not related to the intrinsic circadian rhythm of [Ca^2+^]_c_ in SCN neurons. At this point, however, we need to indicate several other possibilities as well. First of all, our study does not exclude the potential importance of Ca^2+^ spikes, for example, which are related to the modulation of neurotransmitters, for various functions of SCN. Second, it is also possible that there could have been a small subset of cells whose Ca^2+^ spiking rate indeed showed a circadian variation but masked by other non-circadian cells. We also realize that some cortical neurons can support very small and fast Ca^2+^ transients having a bandwidth of about a second or less. Such very fast Ca^2+^ transients could have existed in our experiments but unnoticed due to the limitation of our image acquisition rate (∼1 second). On the other hand, the possible existence of such neurons in SCN has never been addressed nor been documented in the past.

## Supporting Information

Figure S1 Spontaneous Ca^2+^ spiking activities in cultured rat hypothalamic neurons that express yellow cameleon (pNSE/YC). Time series (sampling rate at 1 frame per 1.5 seconds) of the level of [Ca^2+^]_c_ in two different hypothalamic neurons exhibit robust synchronized Ca^2+^ spiking activities.
(0.20 MB DOC)
