# Supplementary material for: Intracellular Calcium Spikes in Rat Suprachiasmatic Nucleus Neurons Induced by BAPTA-Based Calcium Dyes
Source: PLoS One. 2010 Mar 10;5(3):e9634. doi: 10.1371/journal.pone.0009634 (PMC2835761; doi:10.1371/journal.pone.0009634)
Supplement: Figure S1 — Spontaneous Ca2+ spiking activities in cultured rat hypothalamic neurons that express yellow cameleon (pNSE/YC). Time series (sampling rate at 1 frame per 1.5 seconds) of the level of [Ca2+]c in two different hypothalamic neurons exhibit robust synchronized Ca2+ spiking activities. (0.20 MB DOC) [file pone.0009634.s001.doc]

**Supporting Information**


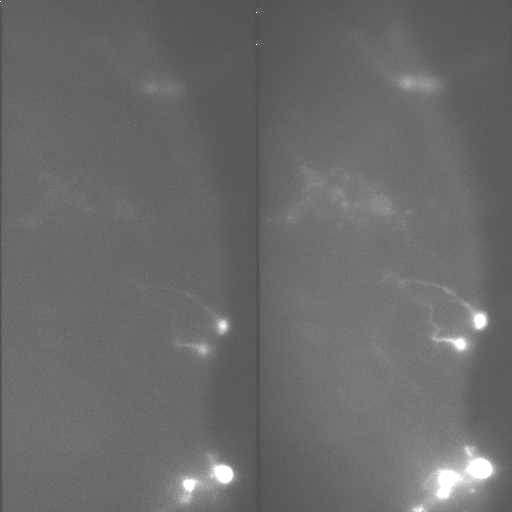

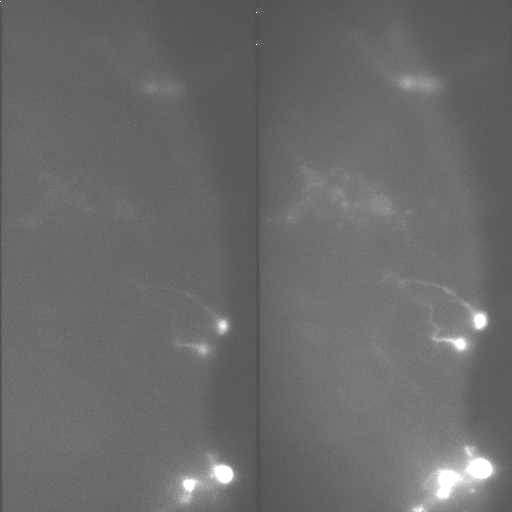


**YFP(F535)**

**CFP(F480)**

**Figure S1** Spontaneous Ca^2+^ spiking activities in cultured rat hypothalamic neurons that express yellow cameleon (pNSE/YC): Time series (sampling rate at 1 frame per 1.5 seconds) of the level of [Ca^2+^]_c_ in two different hypothalamic neurons exhibit robust synchronized Ca^2+^ spiking activities .
